# Supplementary material for: Allomorphy as a mechanism of post-translational control of enzyme activity
Source: Nat Commun. 2020 Nov 2;11:5538. doi: 10.1038/s41467-020-19215-9 (PMC7608592; doi:10.1038/s41467-020-19215-9)
Supplement: Supplementary file 1 — Supplementary Information [file 41467_2020_19215_MOESM1_ESM.pdf]

SUPPLEMENTARY INFORMATION

Allomorphy as a mechanism of post-translational control of enzyme activity

Henry P. Wood[a,1], F. Aaron Cruz-Navarrete[a,1,*], Nicola J. Baxter[a,b,1], Clare R. Trevitt[a], Angus J. Robertson[a,c], Samuel R. Dix[a], Andrea M. Hounslow[a], Matthew J. Cliff[b], and Jonathan P. Waltho[a,b,*]

[a]Krebs Institute for Biomolecular Research, Department of Molecular Biology and Biotechnology, The University of Sheffield, Sheffield, S10 2TN, United Kingdom; [b]Manchester Institute of Biotechnology and School of Chemistry, The University of Manchester, Manchester, M1 7DN, United Kingdom; [c]Present address: Laboratory of Chemical Physics, National Institute of Diabetes and Digestive and Kidney Diseases, National Institutes of Health, Bethesda, Maryland 20892, United States

[1]H.P.W., F.A.C.N. and N.J.B contributed equally to this work

[*]To whom correspondence may be addressed: Prof. Jonathan Waltho and F. Aaron Cruz-Navarrete, Krebs Institute for Biomolecular Research, Department of Molecular Biology and Biotechnology, University of Sheffield, Sheffield, S10 2TN, United Kingdom, +44 114 22717, j.waltho@sheffield.ac.uk, ORCID 0000-0002-7402-5492, facruznavarrete1@sheffield.ac.uk, ORCID 0000-0002-5233-581X

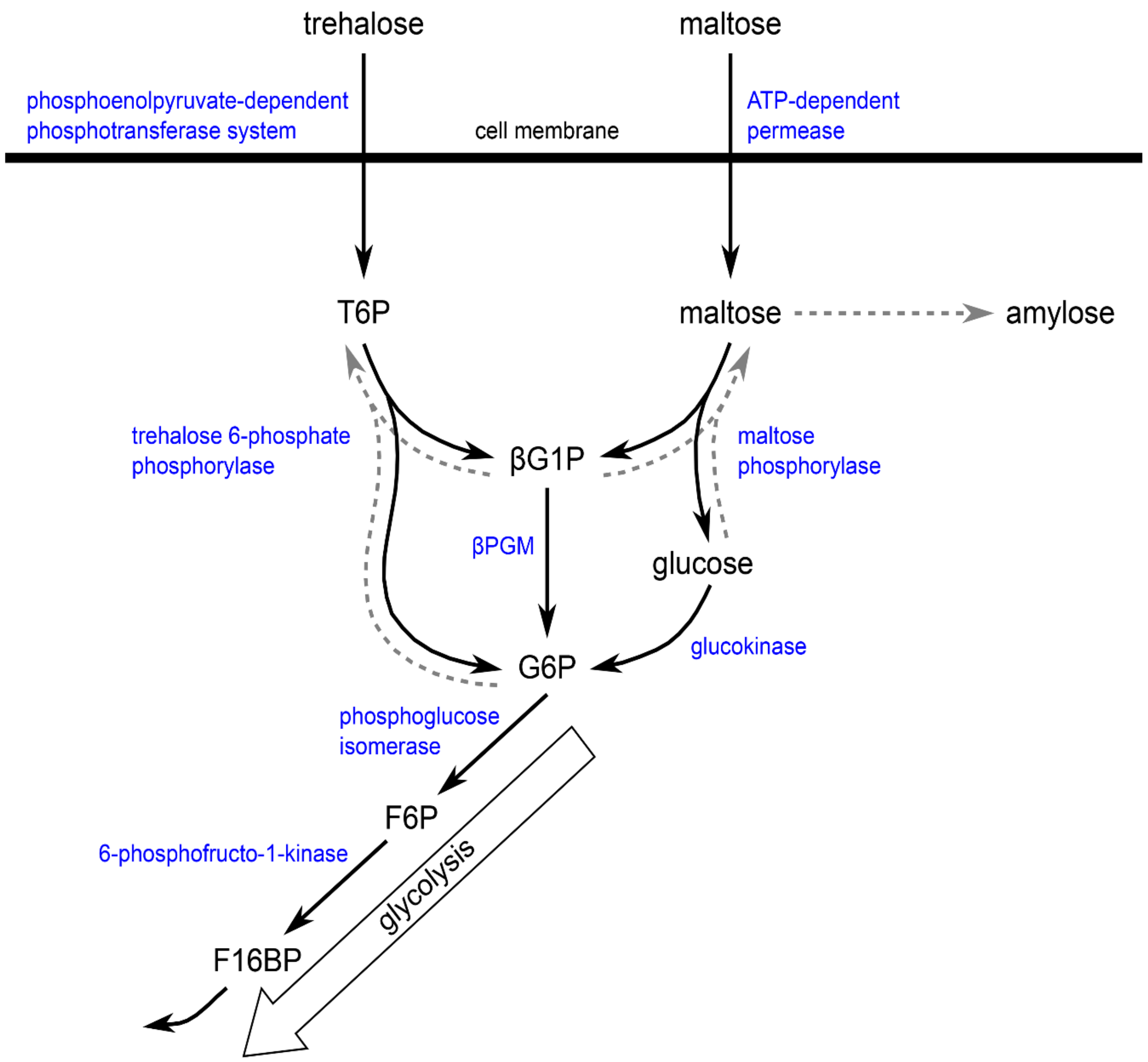

Supplementary Figure 1. Pathways for trehalose and maltose metabolism in *L. lactis*. Trehalose is transported into cells by the phosphoenolpyruvate-dependent phosphotransferase system yielding T6P, which is phosphorolysed by P_i-dependent trehalose 6-phosphate phosphorylase to βG1P and G6P. In contrast, maltose enters cells by the ATP-dependent permease system and is phosphorolysed by the action of P_i-dependent maltose phosphorylase to βG1P and glucose. Glucose is subsequently phosphorylated to G6P by glucokinase and enters glycolysis via fructose 6-phosphate (F6P) and F16BP. βPGM catalyses the isomerisation of βG1P to G6P, allowing complete catabolism of both trehalose and maltose. βPGM deficient *L. lactis* is unable to grow on trehalose and when cultured on maltose (disaccharide composed of α(1→4)-linked glucose units) the cells excrete βG1P into the growth medium and accumulate intracellularly βG1P (~0.7 M), T6P (~2.7 M) and amylose (polysaccharides composed of α(1→4)-linked glucose units). This observation is consistent with both P_i-dependent trehalose 6-phosphate phosphorylase and P_i-dependent maltose phosphorylase operating in the reverse sense (grey dotted arrows) to their physiological roles in wild-type *L. lactis*, resulting in excess βG1P being combined with G6P to form T6P or polymerised as α(1→4) glucose units to form amylose.

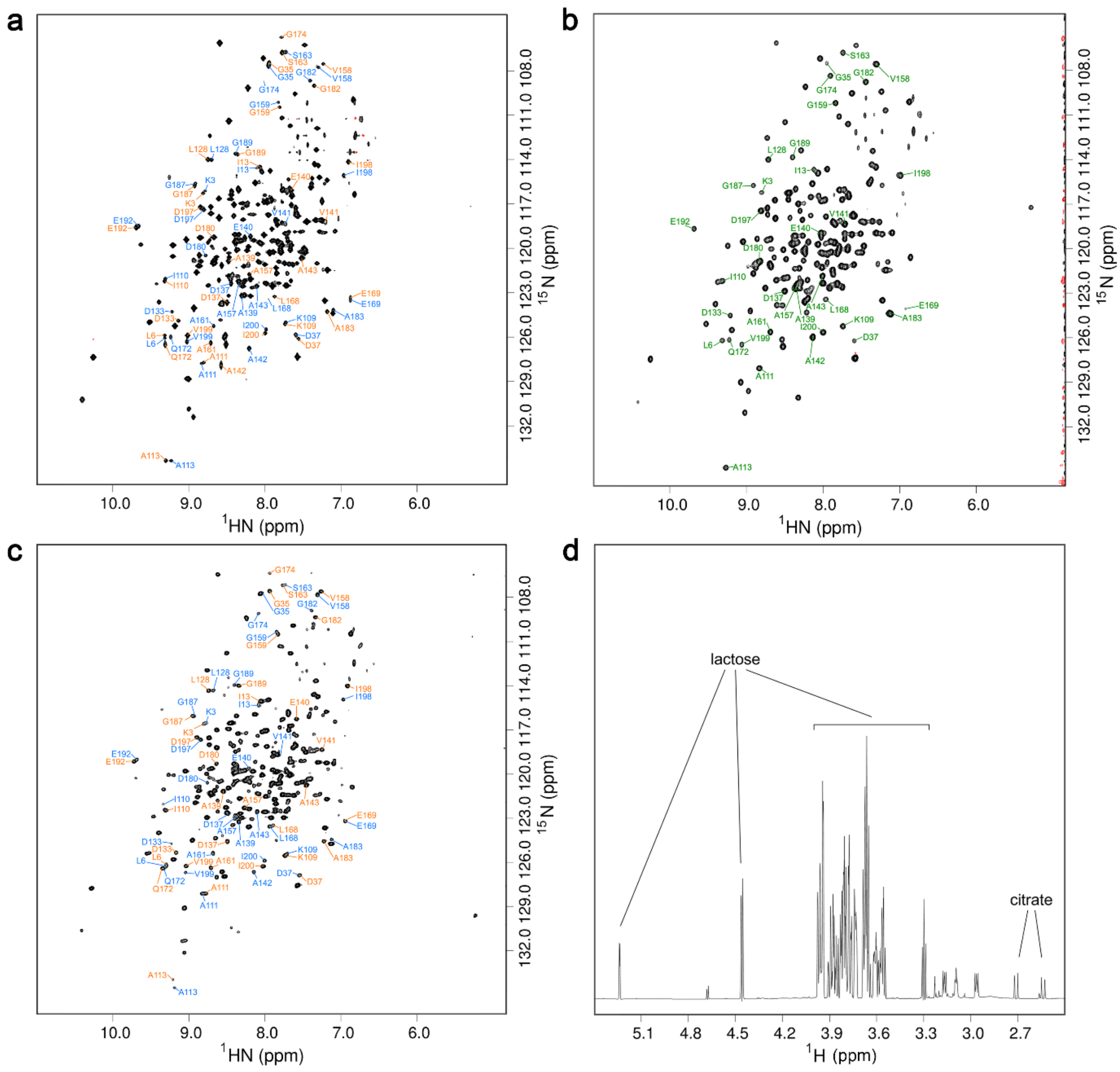

Supplementary Figure 2. Solution behaviour of βPGM. (**a**) $^{1}H^{15}N$-TROSY spectrum of $^{2}H^{15}N^{13}C$-βPGM$_{WT}$ in standard NMR buffer containing 5 mM tris. A selection of well-resolved residues are labelled which populate conformer A (orange labels) and conformer B (blue labels) in slow exchange. (**b**) $^{1}H^{15}N$-TROSY spectrum of $^{2}H^{15}N^{13}C$-βPGM$_{P146A}$ in standard NMR buffer. βPGM$_{P146A}$ populates one conformer and the same selection of residues has been labelled in dark green for comparison. (**c**) $^{1}H^{15}N$-TROSY spectrum of ^{15}N-βPGM$_{WT}$ in filtered milk where both conformers are populated. (**d**) ^{1}H NMR spectrum of ^{15}N-βPGM$_{WT}$ in filtered milk showing the major milk components. The concentrations of lactose and citrate are estimated as 17 mM and 5 mM, respectively.

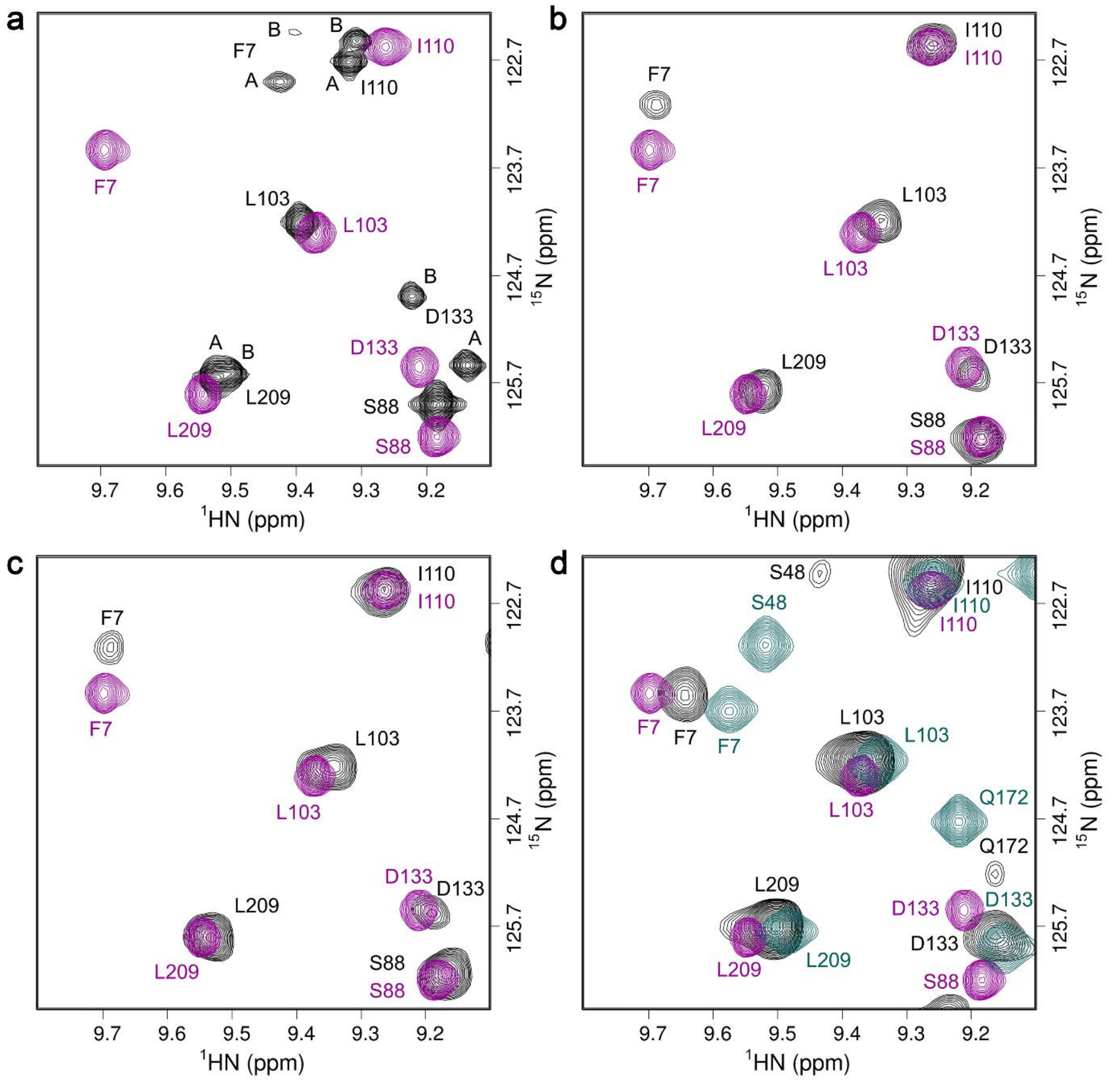

Supplementary Figure 3. Effect of different phosphorylating agents on βPGM$_{WT}$. a–**d**, Comparative overlays of a section of ^{1}H^{15}N-TROSY spectra highlighting the behaviour of residues F7, S48, S88, L103, I110, D133, Q172 and L209. (**a**) βPGM$_{WT}$ (black) populates conformer A and conformer B in slow exchange for a subset of these residues. The addition of 3 mM $BeCl_2$ and 10 mM NH_4F to the βPGM$_{WT}$ sample induces the population of a single βPGM$_{WT}$:BeF_3 complex (magenta) (BMRB 17851), which is an analogue of phosphorylated conformer A (A^P). (**b**) βPGM$_{WT}$ supplemented with AcP (black) populates A^P as the dominant species, which shows a good degree of correspondence with the A^P analogue (magenta). (**c**) βPGM$_{WT}$ supplemented with F16BP (black) populates A^P as the dominant species, which again overlays well with the A^P analogue (magenta). (**d**) βPGM$_{WT}$ supplemented with βG16BP (black) populates a βPGM$_{WT}$:βG16BP complex (A:βG16BP), which shares a better correspondence with the Mg^{2+}-saturated βPGM$_{D10N}$:βG16BP complex (pale blue) (BMRB 27174) than with the A^P analogue (magenta).

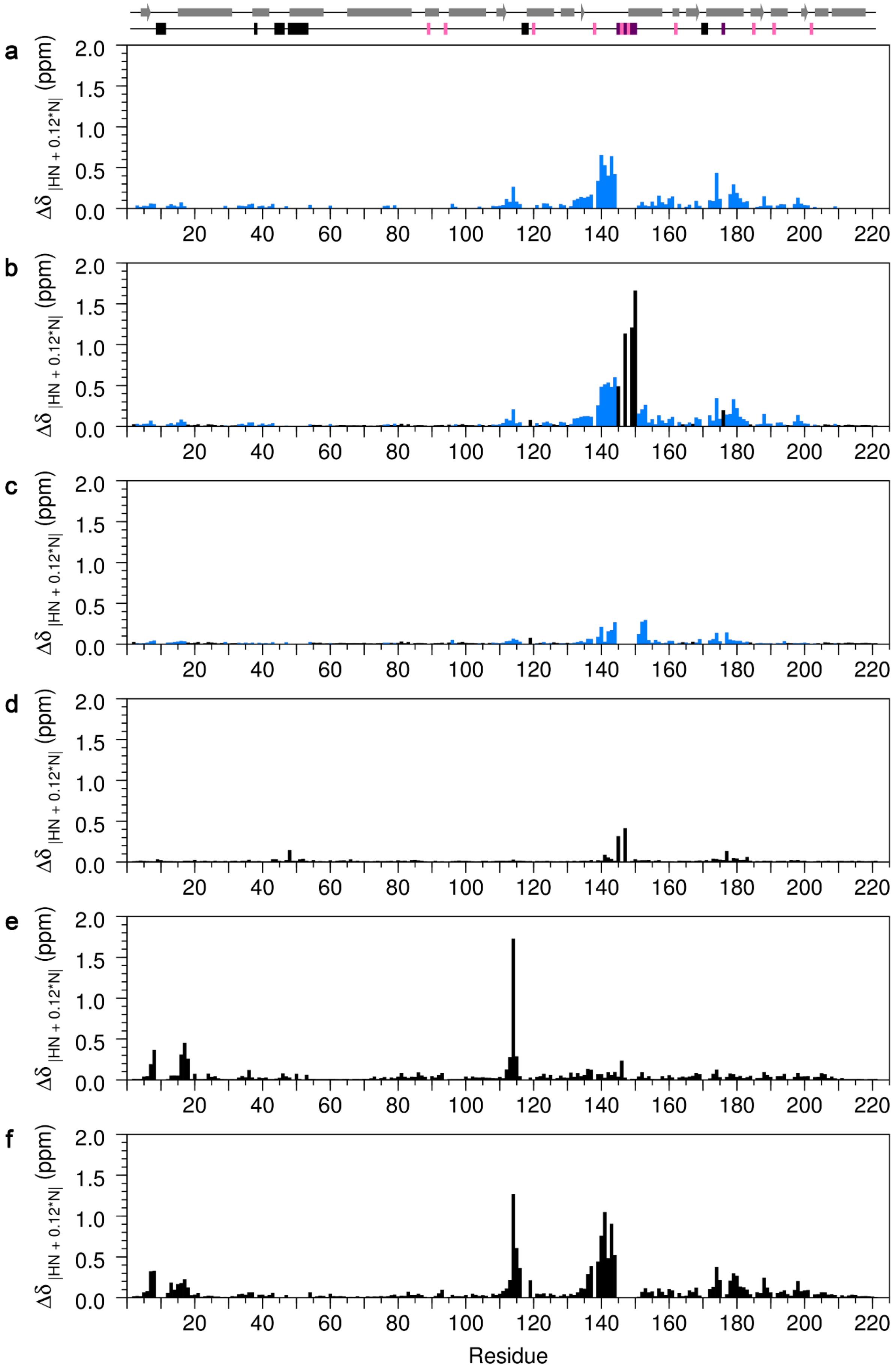
a
b
c
d
e
f
Δδ |HN + 0.12*N| (ppm)
2.0
1.5
1.0
0.5
0.0
20
40
60
80
100
120
140
160
180
200
220
Residue

Supplementary Figure 4. Residue specific backbone amide group chemical shift differences (Δδ) for βPGM$_{WT}$ and βPGM$_{P146A}$. (**a**) Chemical shift differences between conformer A and conformer B. (**b**) Chemical shift differences between conformer A and βPGM$_{P146A}$. (**c**) Chemical shift differences between conformer B and βPGM$_{P146A}$. (**d**) Chemical shift differences between the βPGM$_{WT}$:MgF_3:G6P TSA and the βPGM$_{P146A}$:MgF_3:G6P TSA complexes. (**e**) Chemical shift differences between βPGM$_{P146A}$ and phosphorylated βPGM$_{P146A}$ (B^P). (**f**) Chemical shift differences between conformer B and the βPGM$_{WT}$:BeF_3 complex (A^P analogue). In panels (**b**) and (**c**), bars are coloured blue if residues in (**a**) showed a Δδ value greater than zero, otherwise bars are coloured black. Disregarding the dominant effect of the P146A substitution (large black bars), there are smaller and fewer chemical shift perturbations (blue bars) in (**c**) than in (**b**) suggesting that conformer B adopts a conformation closely similar to βPGM$_{P146A}$. At the top of the panel, secondary structure elements from βPGM$_{WT}$ (PDB 2WHE) are indicated by grey bars (α-helices) and arrows (β-strands). Residues in conformer A and conformer B with missing backbone amide peaks in the $^{1}H^{15}N$-TROSY spectrum of βPGM$_{WT}$ are shown by black rectangles, whereas missing backbone amide peaks in conformer B only are shown by purple rectangles. Proline residues in βPGM$_{WT}$ are denoted by pink rectangles.

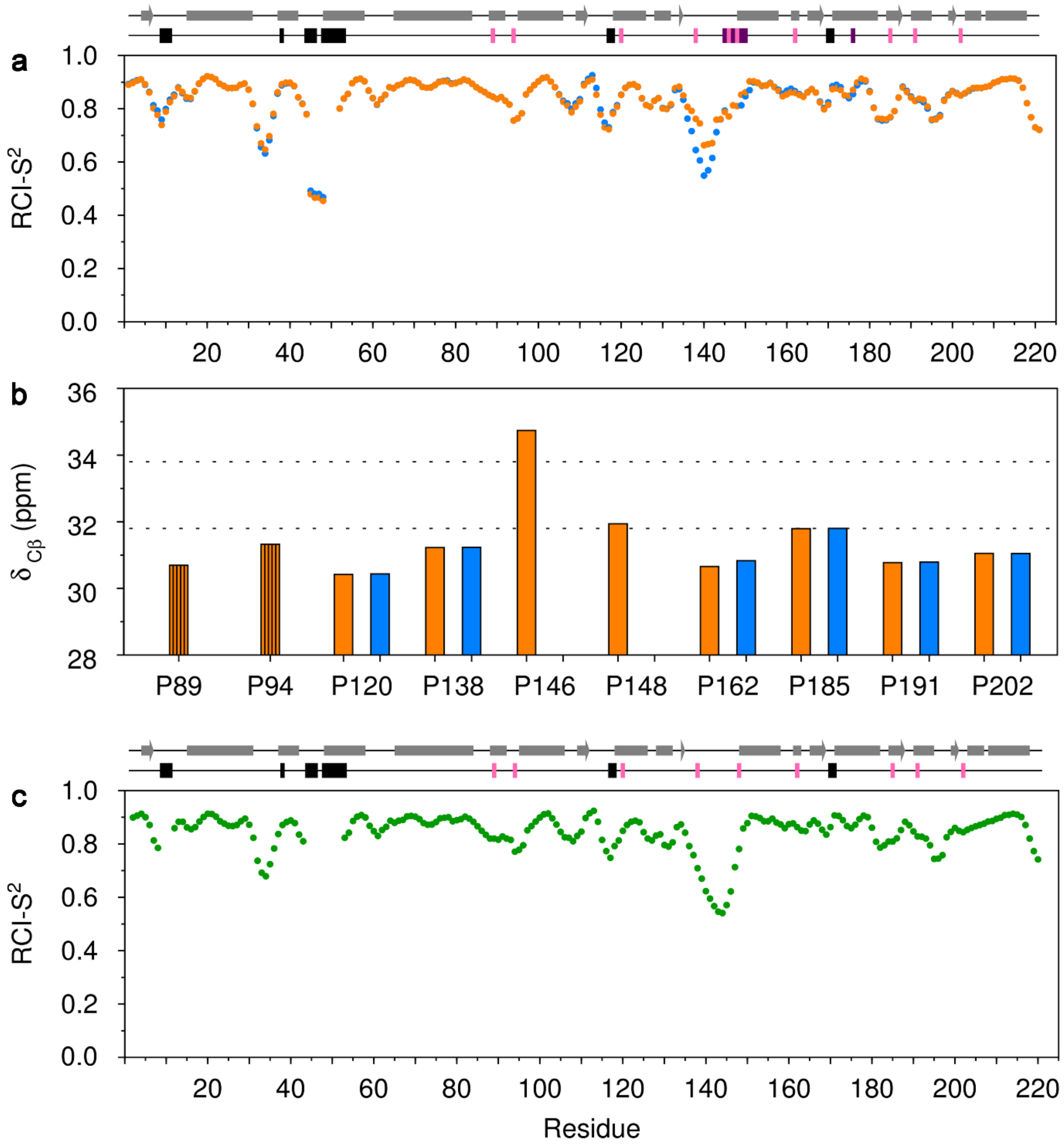

Supplementary Figure 5. Chemical shift analysis of βPGM$_{WT}$ and βPGM$_{P146A}$. (**a**) Random coil index order parameter (RCI-S^2) prediction of conformer A (orange circles) and conformer B (blue circles) obtained using TALOS-N. Secondary structure elements, the extent of assignment and proline residue locations are presented at the top of the panel for βPGM$_{WT}$ as described previously. (**b**) Comparison of assignable proline ^{13}Cβ chemical shifts in βPGM$_{WT}$ for conformer A (orange bars) and conformer B (blue bars). Orange bars with vertical black shading indicate identical chemical shifts for conformer A and conformer B. The ^{13}Cβ resonances of P146 and P148 in conformer B are likely to be broadened beyond detection due to conformation exchange in the K145–I150 region occurring on the millisecond timescale. Dotted horizontal lines represent average proline ^{13}Cβ chemical shift values with *trans* (31.8 ± 1.0 ppm) and *cis* (33.8 ± 1.2 ppm) Xaa-Pro peptide bonds. (**c**) RCI-S^2 prediction of βPGM$_{P146A}$ (dark green circles) obtained using TALOS-N. Secondary structure elements, the extent of assignment and proline residue locations are presented at the top of the panel for βPGM$_{P146A}$ as described previously.

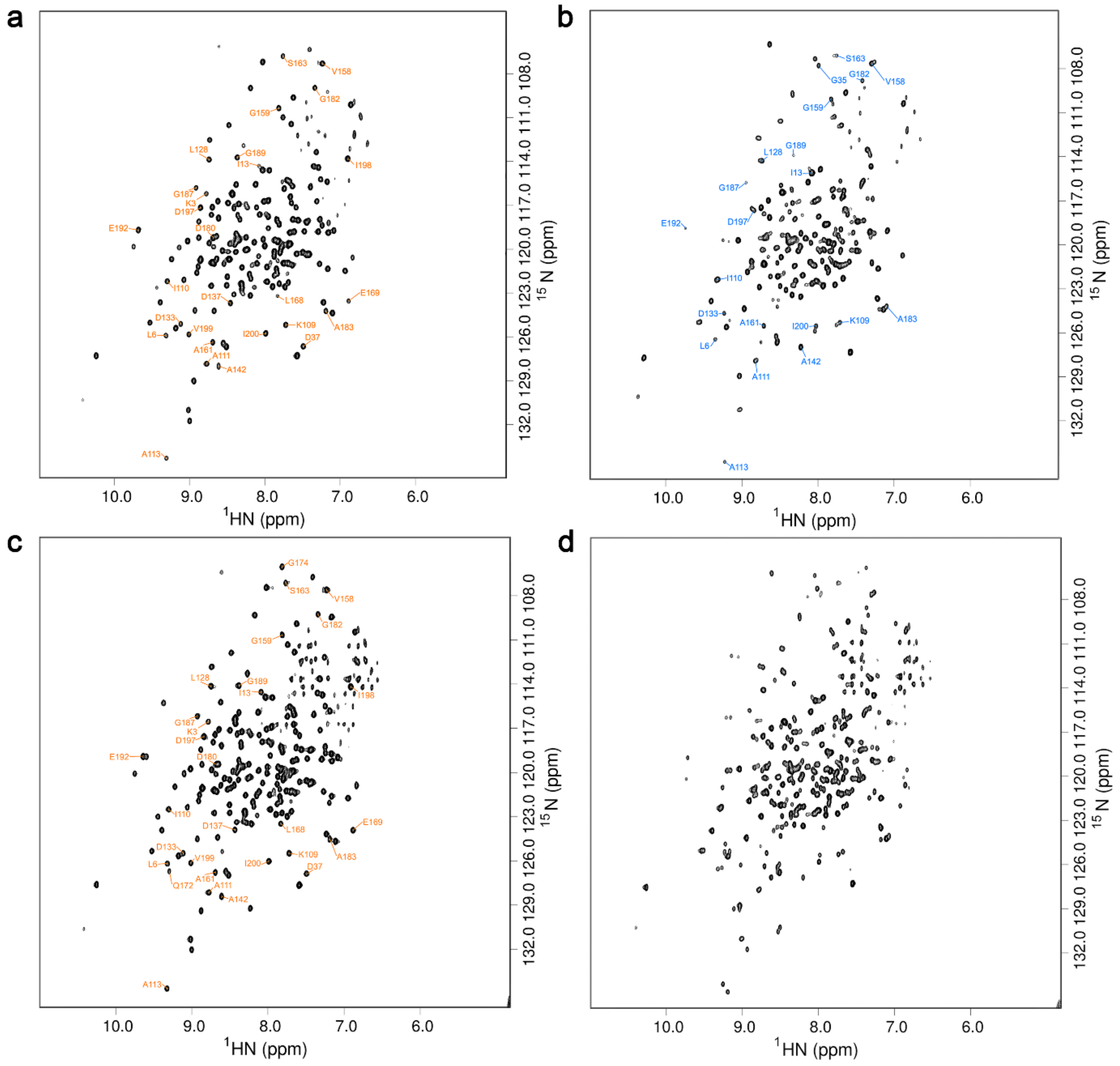

Supplementary Figure 6. Solution behaviour of βPGM$_{WT}$ under variable ion concentrations. (**a**) $^{1}H^{15}N$-TROSY spectrum of ^{15}N-βPGM$_{WT}$ in standard NMR buffer containing 200 mM NaCl. The dominant population of βPGM$_{WT}$ is conformer A. (**b**) $^{1}H^{15}N$-TROSY spectrum of ^{15}N-βPGM$_{WT}$ in deionised water. The dominant population of βPGM$_{WT}$ is conformer B. (**c**) $^{1}H^{15}N$-TROSY spectrum of ^{15}N-βPGM$_{WT}$ in standard NMR buffer containing 100 mM $MgCl_2$. The dominant population of βPGM$_{WT}$ is conformer A. (**d**) $^{1}H^{15}N$-TROSY spectrum of ^{15}N-βPGM$_{WT}$ in Mg^{2+}-free standard NMR buffer. Both conformer A and conformer B are populated.

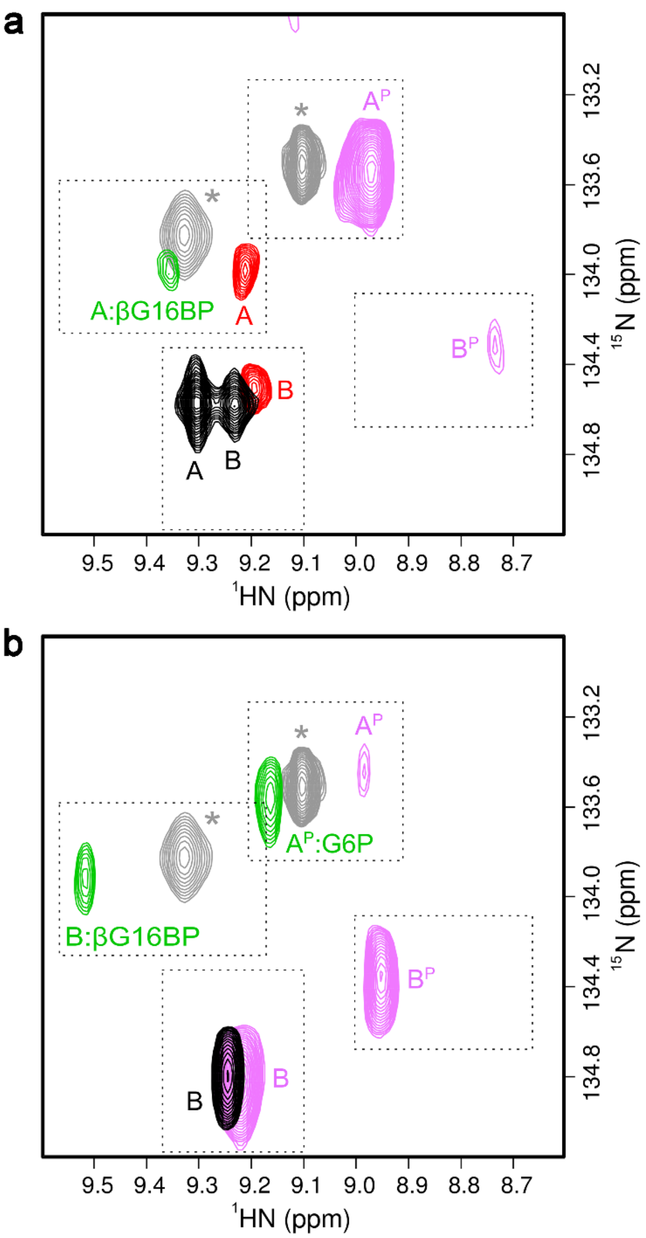

Supplementary Figure 7. Effect of different phosphorylating agents on βPGM. a, **b**, Overlays of a section of $^1H^{15}N$-TROSY spectra for βPGM_{WT} and βPGM_{P146A} highlighting the behaviour of residue A113. (**a**) βPGM_{WT} (black) populates conformer A and conformer B in slow exchange. βPGM_{WT} recorded in filtered milk (red) populates conformer A and conformer B. βPGM_{WT} supplemented with AcP (pink) populates A^P as the dominant species and B^P. βPGM_{WT} supplemented with βG16BP (green) populates an A:βG16BP complex. (**b**) βPGM_{P146A} (black) populates conformer B. βPGM_{P146A} supplemented with AcP (pink) populates conformer B, A^P and B^P. βPGM_{P146A} supplemented with βG16BP (green) populates an A^P:G6P complex and a B:βG16BP complex. Peaks indicated by grey asterisks correspond to the $\beta PGM_{WT}:BeF_3$ complex (grey; δ_N = 133.5 ppm; BMRB 17851), which is an analogue of A^P, and the Mg^{2+}-saturated βPGM_{D10N}:βG16BP complex (grey; δ_N = 133.8 ppm; BMRB 27174), which is a mimic of the A:βG16BP complex, and are shown for comparison.

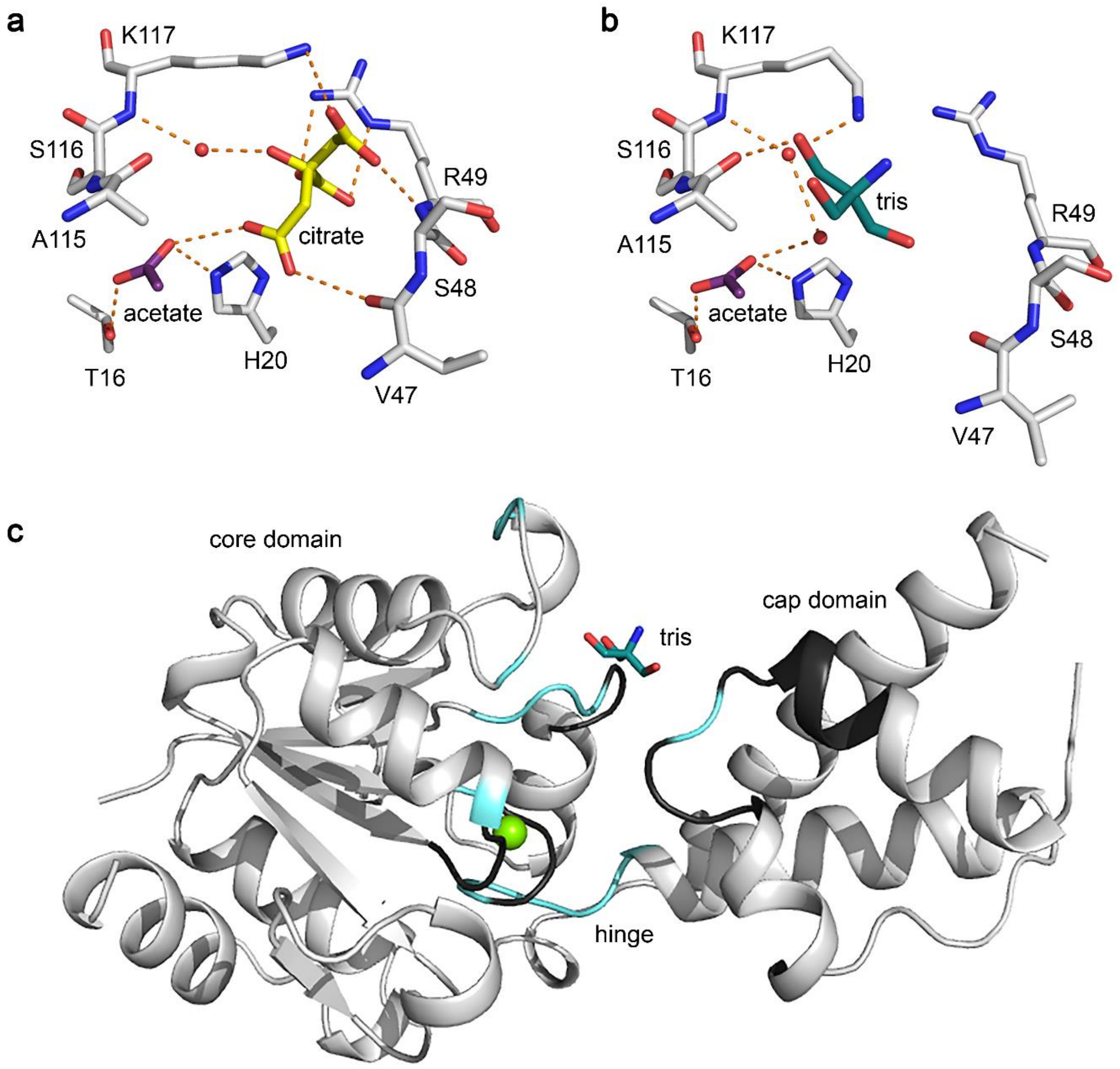

Supplementary Figure 8. Binding of small molecules to βPGM$_{WT}$. a, **b**, Active site coordination for citrate, acetate and tris in the βPGM$_{WT}$:citrate complex (PDB 6YDM). βPGM$_{WT}$ was crystallised in the presence of citrate in a crystallisation buffer containing acetate and tris. Selected active site residues and ligands are shown as sticks with βPGM$_{WT}$ (grey carbon atoms), citrate (gold carbon atoms), acetate (purple carbon atoms) and tris (teal carbon atoms) for both chains of the crystallographic asymmetric unit. Red spheres indicate structural water molecules and orange dashes show probable hydrogen bonds. **c**, Changes in intermediate exchange behaviour on tris binding. Cartoon representation of the βPGM$_{WT}$:citrate complex highlighting the extent of active site residues undergoing intermediate exchange behaviour in the original βPGM$_{WT}$ assignment (without tris; cyan backbone, D8, L9, D10, G11, V12, I13, T14, D15, T16, R38, L44, K45, G46, V47, S48, R49, E50, D51, S52, L53, S114, A115, S116, K117, N118, V141, A142, K145, S171 and Q172; BMRB 7235) and in the assignment of conformer A (with tris; black backbone, L9, D10, G11, R38, L44, K45, G46, S48, R49, E50, D51, S52, L53, K117, N118, D170 and S171; BMRB 28095). The coordination of tris (teal carbon atoms) in the active site cleft perturbs the exchange behaviour for some loop residues, with the result that the corresponding resonances are no longer broadened beyond detection and can therefore be assigned. The catalytic Mg^{2+} ion is shown as a green sphere.

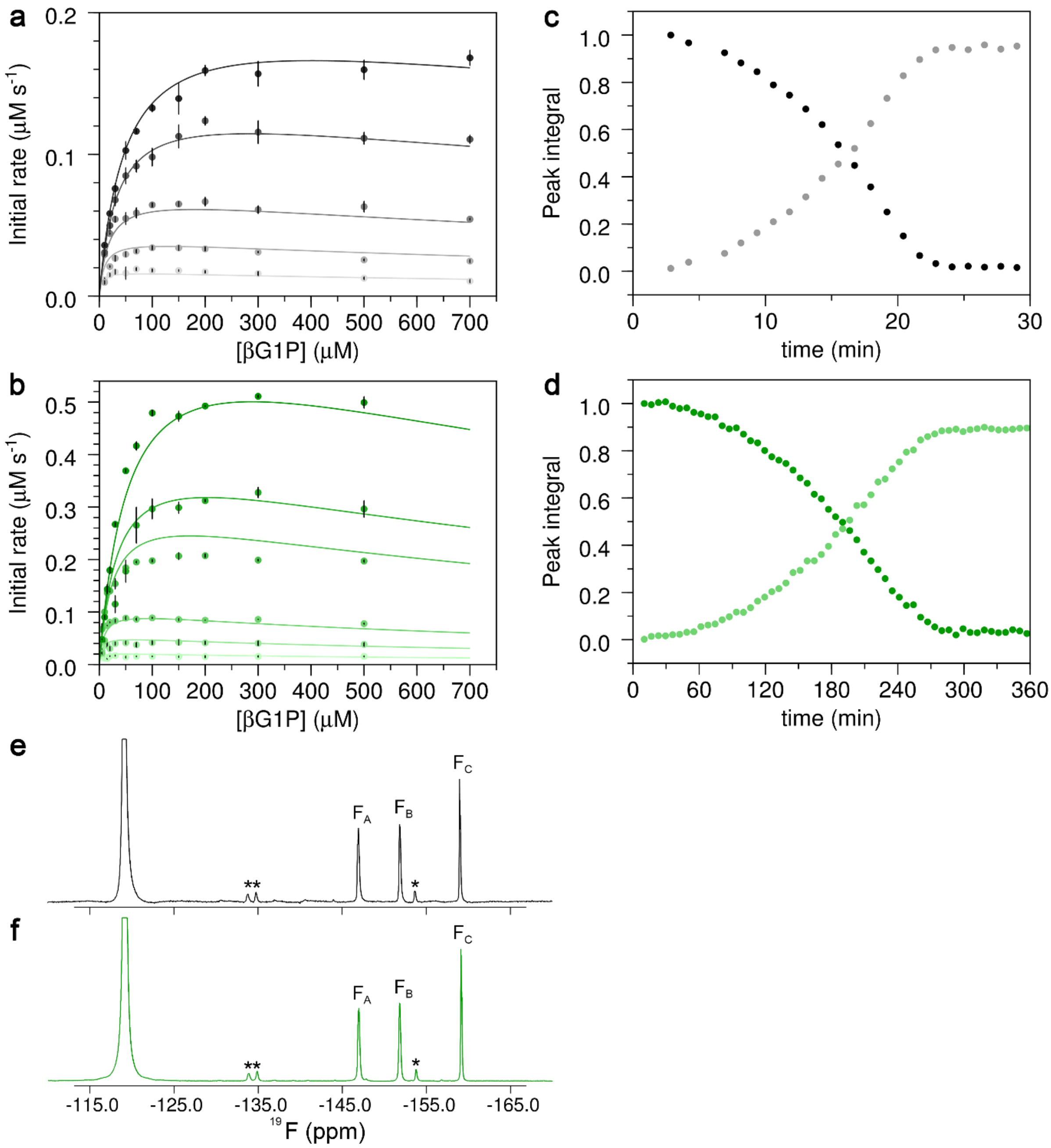

Supplementary Figure 9. Activity of βPGM$_{WT}$ and βPGM$_{P146A}$. **a**, **b**, Initial rate measurements for βPGM$_{WT}$ and βPGM$_{P146A}$ using the coupled assay. The solid lines represent a global fit of the data to Equation 1 and vertical black lines indicate standard error of the mean of three replicate measurements. (**a**) βPGM$_{WT}$ initial rate measurements at a range of βG1P concentrations (10, 20, 30, 50, 70, 100, 150, 200, 300, 500, 700 µM) and βG16BP concentrations (0.4, 1, 2, 5, 10 µM, grey gradient increasing with concentration). (**b**) βPGM$_{P146A}$ initial rate measurements at a range of βG1P concentrations (5, 10, 15, 20, 30, 50, 70, 100, 200, 300, 500 µM) and βG16BP concentrations (2, 5, 10, 35, 50, 100 µM, green gradient increasing with concentration). **c**, **d**, Reaction kinetics monitored by ^{31}P NMR spectra for βPGM$_{WT}$ (grey tones) and βPGM$_{P146A}$ (green tones) for the equilibration of βG1P with G6P in standard kinetic buffer. The reactions were initiated by the addition of 20 mM AcP. Normalised integral values for the βG1P peak (black / dark green) and the G6P peak (grey / light green) are plotted as a function of time. **e**, **f**, ^{19}F NMR spectra of the βPGM:MgF$_3$:G6P TSA complexes formed using either βPGM$_{WT}$ (black) or βPGM$_{P146A}$ (green) in standard NMR buffer, supplemented with 15 mM NaF and 10 mM G6P. Chemical shifts are given in ppm for each ^{19}F resonance: βPGM$_{WT}$:MgF$_3$:G6P TSA complex (F$_A$

= –146.9, F_B = –151.9 and F_C = –159.0) and βPGM$_{P146A}$:MgF$_3$:G6P TSA complex (F_A = –147.0, F_B = –151.8 and F_C = –159.2). Resonances indicated by black asterisks correspond to an alternative conformation of the βPGM:MgF$_3$:G6P TSA complexes. Free $F^–$ resonates at –119.1 ppm and the full peak intensity has been truncated for clarity.

Supplementary Table 1. Data collection, data processing and refinement statistics for the βPGM complexes

Complex	βPGM_{WT}:citrate	βPGM_{WT}	βPGM_{P146A}	$\beta PGM_{P146A}:MgF_3$:G6P
**PDB Code**	PDB 6YDM	PDB 6YDL	PDB 6YDK	PDB 6YDJ
**Crystallisation conditions**	0.6 mM βPGM_{WT}<br>50 mM citrate	0.6 mM βPGM_{WT}	0.5 mM βPGM_{P146A}	0.4 mM βPGM_{P146A}<br>10 mM G6P<br>15 mM NaF
**Crystal morphology**	Rod shaped crystals	Rod shaped crystals	Rod shaped crystals	Large plate crystals
**Wavelength (Å)**<br>**Beamline, Facility**	0.97179<br>Beamline i03, DLS	0.92819<br>Beamline i04-1, DLS	0.97950<br>Beamline i04, DLS	0.91587<br>Beamline i04-1, DLS
**Resolution (Å)** [1]	46.57 – 2.10<br>(2.16 – 2.10)	44.65 – 1.52<br>(1.56 – 1.52)	43.95 – 2.02<br>(2.05 – 2.02)	54.25 – 1.04<br>(1.06 – 1.04)
**Space group**	$P2_12_12_1$	$P2_12_12_1$	$P2_12_12_1$	$P2_12_12_1$
**Cell dimensions:**<br>**a, b, c (Å)**<br>**α, β, γ (°)**	 53.1, 76.6,117.3<br>90.0, 90.0, 90.0	 53.3, 54.1, 81.9<br>90.0, 90.0, 90.0	 53.3, 56.2, 77.7<br>90.0, 90.0, 90.0	 37.1, 54.3, 104.3<br>90.0, 90.0, 90.0
**Total reflections** [1]	239876 (15971)	264843 (16619)	103063 (2568)	680305 (21980)
**Unique reflections** [1]	27995 (2095)	36815 (2658)	15683 (686)	101730 (5021)
**Multiplicity** [1]	8.6 (7.6)	7.2 (6.3)	6.6 (3.7)	6.7 (4.4)
**Completeness (%)** [1]	97.5 (90.1)	99.3 (99.3)	98.8 (88.9)	99.9 (99.3)
**$\langle I/\sigma I \rangle$** [1]	13.7 (3.7)	18.8 (1.3)	14.4 (1.2)	12.4 (1.1)
**Wilson B factor ($Å^2$)**	24.2	21.2	32.2	7.4
**R_{merge}** [1]	0.093 (0.536)	0.044 (1.190)	0.075 (0.953)	0.067 (1.138)
**R_{pim}** [1]	0.033 (0.198)	0.019 (0.561)	0.031 (0.506)	0.028 (0.609)
**CC-half** [1]	0.999 (0.899)	1.000 (0.536)	0.999 (0.556)	0.999 (0.544)
**Molecular replacement model**	PDB 2WHE	PDB 2WHE	PDB 2WHE	PDB 2WF5
**R_{factor}**	0.230	0.183	0.214	0.149
**R_{free}**	0.290	0.216	0.253	0.169
**Number of atoms:**<br>**Protein**<br>**Ligands**<br>**Metal ions**<br>**Water**	 3379<br>38<br>2<br>187	 1733<br>0<br>1<br>148	 1708<br>0<br>1<br>36	 1704<br>29<br>2<br>209
**Protein residues**	438	219	221	218
**RMS deviations:**<br>**Bonds (Å)**<br>**Angles (°)**	 0.0089<br>1.482	 0.0145<br>1.497	 0.0125<br>1.522	 0.0093<br>1.473
**Average B factors ($Å^2$)**<br>**Main chain**<br>**Side chains**<br>**Ligands**<br>**Metal ions**<br>**Water**	 32<br>36<br>50<br>27<br>33	 25<br>29<br>N/A<br>27<br>36	 15<br>27<br>N/A<br>44<br>39	 11<br>13<br>10<br>10<br>24
**Ramachandran analysis**<br>**Favoured/allowed (%)**<br>**Disallowed (%)**<br>**Favoured rotamers (%)**<br>**Poor rotamers (%)**	 97.7<br>0.0<br>94.1<br>1.10	 98.6<br>0.0<br>96.2<br>0.54	 97.7<br>0.0<br>96.7<br>1.11	 97.7<br>0.0<br>95.1<br>0.55
**MolProbity score**	1.29<br>(99th percentile,<br>2.10 ± 0.25 Å)	0.93<br>100th percentile,<br>1.52 ± 0.25 Å)	0.98<br>(100th percentile,<br>2.02 ± 0.25 Å))	0.66<br>(99th percentile,<br>1.04 ± 0.25 Å)

[1] Values for the higher resolution shell are in parentheses
